# The Importance of Sample Size for Reproducibility of tDCS Effects

**DOI:** 10.3389/fnhum.2016.00453

**Published:** 2016-09-13

**Authors:** Tamas Minarik, Barbara Berger, Laura Althaus, Veronika Bader, Bianca Biebl, Franziska Brotzeller, Theodor Fusban, Jessica Hegemann, Lea Jesteadt, Lukas Kalweit, Miriam Leitner, Francesca Linke, Natalia Nabielska, Thomas Reiter, Daniela Schmitt, Alexander Spraetz, Paul Sauseng

**Affiliations:** Department of Psychology, Ludwig-Maximilians-Universität MünchenMunich, Germany

**Keywords:** anodal tDCS, cathodal tDCS, choice reaction time task, M1, open science, response times

## Introduction

Cheap, easy to apply, well-tolerable, with the potential of altering cortical excitability, and for testing causalities—these are attributes that have made transcranial direct current stimulation (tDCS) a highly popular research tool in cognitive neuroscience. Since its reintroduction over 15 years ago by Nitsche and Paulus (2000), the number of publications reporting tDCS results has risen exponentially (a Scopus® literature search indicates over 500 such journal articles published in 2015 alone). Recently however, the efficacy of tDCS to alter cognitive performance has been called into question, in particular among healthy participants, but also in certain clinical samples (Horvath et al., 2015; Hill et al., 2016; Mancuso et al., 2016). A number of empirical studies reported not having been able to detect any facilitatory effects of anodal tDCS or inhibitory effects of cathodal tDCS on various cognitive processes (e.g., Wiethoff et al., 2014; Minarik et al., 2015; Sahlem et al., 2015; Horvath et al., 2016; Vannorsdall et al., 2016). In fact, in a recent meta-analysis Horvath et al. (2015) argue that in young, healthy participants there is no effect of tDCS on cognition whatsoever, whereas other meta- analyses do find specific modulation of cognitive processes by tDCS; however, these effects seem to be rather weak (Hill et al., 2016; Mancuso et al., 2016). In a recent commentary the field of tDCS research was even called a research area of bad science (Underwood, 2016) in desperate need of further meticulous evaluation. Although there seems to be some inconsistency of effects there is also current work by Cason and Medina (2016) suggesting no evidence for p-hacking (strategic testing and analysis procedures to increase likelihood of obtaining significant effects) in tDCS research. However, Cason and Medina (2016) find average statistical power in tDCS studies to be below 50%. Therefore, one potential reason for the reported inconsistencies might be that sample size is usually very small in most tDCS studies (including those from our research group). Whilst this issue is not specific to tDCS studies (in fact Button et al., 2013 estimate the median statistical power in neuroscience in general being only 21%), it could lead to weaker effects often not being detected, and consequently meta- analyses suggesting small or no efficacy of tDCS. In addition, the assessment of the real effect of tDCS is further complicated by potential publication bias (file drawer problem) leading to over-reporting significant tDCS findings. That is, a publication bias favoring studies with significant effects might lead to an inflation of the reported efficacy of tDCS. Thus, depending on which studies are included in systematic reviews and meta- analyses (i.e., findings published in peer-reviewed journals; unpublished nil-effects; nil-effects reported as an additional finding in papers with the actual focus on another, significant, effect, etc.), small sample size in tDCS research could lead to both under—and overestimation of tDCS efficacy. Some current meta- analyses (e.g., Mancuso et al., 2016), however, include an estimation of publication bias (e.g., using the “trim and fill” procedure in which funnel plots are used for determining whether there is a bias toward studies with significant effects in the literature included in the meta- analysis); and overall effect size can then be adjusted accordingly. Taking publication bias into account it becomes evident that efficacy of tDCS is rather weak (Mancuso et al., 2016).

As indicated by quite some inconsistency in literature on the efficacy of the stimulation, the field of tDCS research is clearly struck by the replication crisis that we also find in psychology and neurosciences in general (Button et al., 2013; Open Science Collaboration, 2015). But how to estimate efficacy of tDCS, if it is not clear, how many unsuccessful experimental attempts end up in the file drawer? As discussed above, one possibility is to adjust for publication bias in meta- analyses. Another strategy is pre-registering tDCS studies and reporting their outcome, independent of whether the results are significant or not—be it in peer reviewed journals or platforms such as the Open Science Framework (https://osf.io); this can result in more accurate estimates of efficacy. Moreover, allowing open access to the acquired data (open data) offers the opportunity that researchers could pool raw data from experiments with small samples but similar experimental designs. By doing so, they overcome the problem of under-powering, an issue that seems so fundamental in tDCS research.

Therefore, to investigate the effect of sample size on tDCS efficacy and to contribute to increased research transparency we designed a simple, pre-registered study (https://osf.io/eb9c5/?view_only=2743a0c4600943c998c2c37fbfb25846) with a sufficiently large number of young, healthy volunteers estimated with *a priori* power analysis. Furthermore, we make all the acquired data publicly available. In a choice reaction time task (CRT) participants underwent either anodal or cathodal tDCS applied to the sensorimotor cortex. Jacobson et al. (2012) suggest that for the motor domain with tDCS over sensorimotor cortex anodal-excitation and cathodal-inhibition effects (AeCi) are quite straight forward, whereas in other cognitive domains AeCi effects seem not particularly robust. Since we stimulated the sensorimotor cortex we decided to contrast anodal with cathodal tDCS (instead of sham stimulation) for obtaining the largest possible effect. We expected anodal stimulation to result in faster response times compared to cathodal tDCS in accordance with findings by Garcia-Cossio et al. (2015). To demonstrate the importance of sample size for finding the predicted effect, random samples of different sizes were drawn from the data pool and tested statistically. This way the probability of identifying the predicted effect was obtained as a function of sample size.

## Materials and methods

A power analysis (Faul et al., 2007) for an independent-sample *t*-test was conducted assuming one-tailed testing, an effect size of *d* = 0.6, 80% power and alpha error probability of α = 0.05. This analysis suggested a total sample size of at least 72 participants.

We tested 75 participants, randomly assigned to either anodal tDCS (24 female and 14 male; mean age: 22 year [SEM = 0.61]) or cathodal tDCS (19 female and 18 male; mean age: 22.8 year [SEM = 0.59]). The groups did not differ in age [*t*_(73)_ = 0.89, *p* = 0.38] or gender distribution (χ^2^ = 1.07, *p* = 0.30). All volunteers were right handed, had normal or corrected to normal vision, and did not meet any exclusion criteria for tDCS (Nitsche et al., 2003; Woods et al., 2016). The study was approved by the local ethics committee and conducted according to the Declaration of Helsinki.

Volunteers performed a CRT task. In each trial either a diamond (requiring left button press) or a square (requiring right button press) was presented in the center of a monitor for 100 ms followed by an inter-trial interval with a length of 1700–2100 ms. The experiment started with a 2-min training block comprising 60 trials. This was followed by a baseline block of 120 trials. Then tDCS was started. After 4 min of stimulation another block of 120 trials was performed while tDCS continued until the end of the experiment.

In a between-subjects design either anodal or cathodal tDCS was delivered to the left motor cortex. The stimulation electrode was applied with its center at 10-20-electrode position C3. The return electrode was placed above the right orbita. tDCS was delivered at 1 mA (with a ramp-up time of 20 s and ramp-down of 2 s) over 8 min in total. Since we conducted the task during tDCS and did not test during a potential after-effect of tDCS we assumed a total stimulation time of 8 min to be sufficient. We, however, cannot exclude that longer stimulation duration might produce a larger effect. A TCT tDCS stimulator (TCT Research Limited, Hong Kong) with 35 cm^2^ large sponge electrodes soaked in saline water was used.

For each participant the median RT of correctly responded trials only was calculated for the baseline block and the stimulation block separately. Then RT differences between the stimulation block and the baseline block were obtained (ΔRT) and used for statistical analysis. Percentage of correctly responded trials was used as a measure of task accuracy.

## Pre-registration, open data and open material

This is a pre-registered study. The project description is available on open science framework (https://osf.io/eb9c5/?view_only=2743a0c4600943c998c2c37fbfb25846). Presentation® raw data log files as well as processed data for each volunteer are accessible here: https://osf.io/xnyar/?view_only=2743a0c4600943c998c2c37fbfb25846. Data documentation can also be found there. We also provide open access to a Matlab® script we used to draw random samples of different size and perform t-statistics on them, the required input files for this procedure, and its results (https://osf.io/eurcq/?view_only=57080ff7b15f492fa1c343e26c113133). Open material (Presentation® code and stimulus material) can be found here: https://osf.io/nw2hj/?view_only=e083cfb8fc81424ca02e916b40c0378c.

## Results

**Figure 1:**
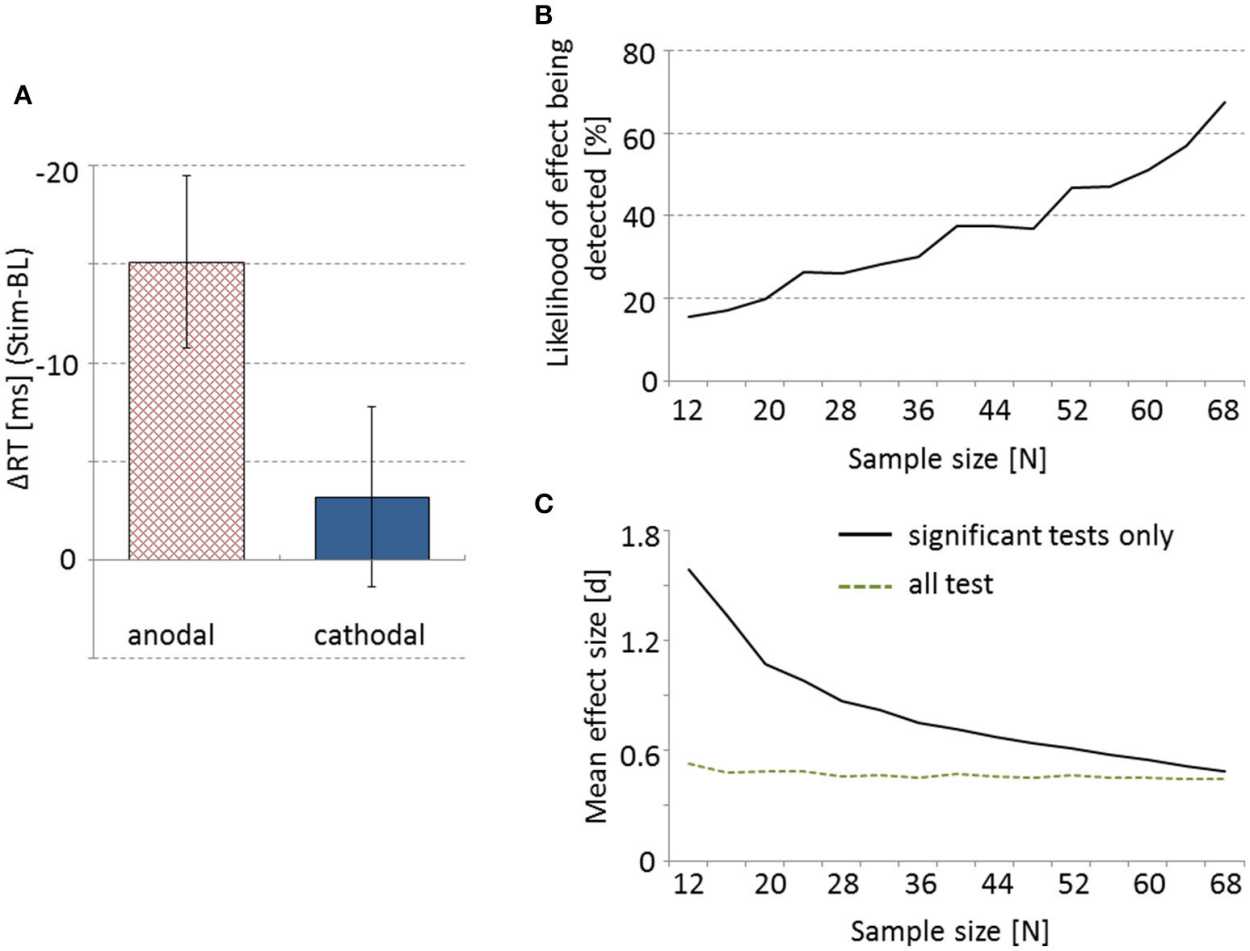
**Anodal tDCS leads to significantly larger response time reduction from baseline to post-stimulation compared to cathodal stimulation (A)**. Error bars represent SEM. **(B)** Percentage of 500 random draws at given sample sizes in which the effect shown in **(A)** can be obtained. **(C)** The solid line depicts mean effect size d as a function of sample size for randomizations where a significant effect was obtained only. The dotted line represents effect size d averaged over all the 500 random draws at each sample size.

All requirements for parametric testing were met. As predicted, ΔRT was significantly different between anodal and cathodal tDCS [*t*_(73)_ = −1.91, *p* = 0.03 [one-tailed], *d* = 0.45], with anodal stimulation resulting in faster RTs than cathodal tDCS (see Figure 1A). Additional one-sample *t*-tests indicate that compared to baseline anodal tDCS resulted in significantly faster RTs [*t*_(37)_ = −3.49, *p* = 0.001, *d* = 1.15], whereas no such effect was obtained for cathodal stimulation [*t*_(36)_ = −0.71, *p* = 0.48, *d* = 0.27]. RTs of the baseline block did not differ significantly between the two groups [*t*_(730_ = 0.66, *p* = 0.51, *d* = 0.15].

To demonstrate the drastic effect of sample size on the probability of detecting the above mentioned effect on RTs we drew random samples of different sizes from our pool of participants and conducted the statistics as described above. For each sample size between 12 and 68 participants we drew 500 samples and each time performed a *t*-test comparing anodal and cathodal stimulation. As depicted in Figure 1B, with very small sample sizes we found statistically significant effects in less than 20% of the cases. Notably, with a sample size of 12 the opposite significant effect was found in two instances, i.e., faster RTs in cathodal than anodal tDCS. Even with a sample size of 60 participants, the hypothesized effect was detected only in 51% of the cases.

For each sample size the average effect size d was calculated over only those randomizations that showed a significant effect (e.g., the 15.4% of tests for sample size 12, etc.). Average effect size as a function of sample size is depicted in Figure 1C, with very high effect sizes for small samples (that still led to a significant *t*-test) to medium and small effect sizes with samples larger than 60 participants. When we, however, averaged all 500 obtained effect sizes for each sample size, independent of whether the *t*-test was significant or not, we observed a relatively stable mean effect size around *d* = 0.45—a value fairly representative for the real effect in our data (Figure 1C).

Task accuracy data were not normally distributed; therefore a Mann-Whitney-*U*-test was performed. There was no significant effect of tDCS on task accuracy (*z* = −0.34, *p* = 0.37 [one-tailed], *r* = 0.04).

## Discussion

Our results suggest that tDCS over the sensorimotor cortex modulates response times in a CRT task, with anodal tDCS leading to faster RTs compared to cathodal stimulation. It is important to point out, however, that in this study there was no sham stimulation condition included. Hence and also because a training effect could have distorted RT differences from baseline to stimulation conditions, it is impossible to conclude whether only anodal, only cathodal or both stimulation conditions have an impact on cognition. This is despite anodal tDCS leading to a significant reduction in RTs compared to baseline, while cathodal tDCS showing no difference to baseline. Jacobson et al. (2012) found that for stimulation of the motor cortex effects of excitation by anodal tDCS and inhibition by cathodal stimulation are fairly consistent. Therefore, it is rather unlikely that in this study a larger overall effect would have been obtained if only one active stimulation condition was compared to sham.

Most importantly, however, here we demonstrate how essential a sufficiently large sample size is for finding an effect of tDCS on cognitive processes in healthy, young participants. With sample sizes of up to 20 participants we found a significant modulation of RTs by tDCS in less than 20% of tests. This very nicely resembles the anecdotal impression (from personal communication with colleagues) of only roughly every fifth tDCS experiment with small sample sizes finding a predicted effect. Even a sample size of 60 participants produced the significant difference between anodal and cathodal tDCS in only 51% of randomizations. That might be somewhat surprising, since in this research field such a sample size would probably be considered as rather large. However, an *a priori* power-analysis suggested a sample size of 72 participants in order to achieve 80% probability of detecting an effect with an effect size of *d* = 0.6. The actual effect size that we found in our experiment was only at *d* = 0.45. This means that *post-hoc* even with our sample of 75 participants the experiment was slightly under-powered. Here, however, it should be noted that sufficient sample size might be substantially smaller in within-subjects designs.

Under-powered tDCS studies might have a range of negative consequences. First, the number of false negatives can be increased. Meta- analyses, therefore, could underestimate the efficacy of tDCS, based on the number of reported false negative results. Moreover, there might be more false positive results, detecting non-existing effects by chance. In our randomization procedure we found significant but reversed effects in a few very small samples (12 participants). This would lead to irreproducible results further counteracting efficacy estimates in meta- analyses. Finally, only fairly vast effects stand a chance of becoming statistically significant in small samples (see Figure 1C). Due to publication bias, studies reporting significant results are more likely to become published in peer-reviewed journals. On a single study level this can lead to an overestimation of effect sizes. Since *a priori* power-analyses assuming these large effect sizes will erroneously suggest relatively small sample sizes, this file drawer problem can have negative impact on the planning of follow-up experiments and replication attempts. If, however, studies are pre-registered and data are open access, failed attempts can be taken into account. As suggested in our analysis the mean effect size over all the attempts (successful as well as unsuccessful ones) is a relatively stable measure of the true effect, even in small samples. Alternatively, meta- analyses correcting for publication bias (e.g., applying “trim and fill” procedures; see Mancuso et al., 2016) give a more accurate measure of overall effect size as well.

Although we only investigated effects of tDCS delivered to sensorimotor cortex on performance in the motor domain, it is plausible that studies using other stimulation parameters and other cognitive tasks are similarly affected by sample size. Since Jacobson et al. (2012) suggest that in cognitive domains other than the motor domain tDCS does not show these clear AeCi effects it is likely that tDCS studies investigating non-motor cognition might even be more affected by sample size issues than demonstrated in the current study. Additionally, task difficulty might influence tDCS efficacy in higher cognitive functions as well (Jones and Berryhill, 2012).

Open data can further contribute to a better evaluation of tDCS efficacy. The pooling of data from several studies with small samples but similar experimental designs will create large data sets that allow the estimation of efficacy much more precisely. This way, small tDCS data sets can best contribute to accurate and rigorous testing of the method. Another advantage is that accessible data can be re-analyzed with statistical methods that are more robust against smaller sample sizes. For instance, the replication rate in psychological studies seems higher than originally reported (Open Science Collaboration, 2015) when Bayes statistics are used for data analysis (Etz and Vandekerckhove, 2016).

## Conclusion

We conclude and recommend that tDCS studies need to be planned more carefully, particularly when it comes to estimation of the to-be-tested sample size. *A priori* power analyses are an important tool for doing so. While due to publication bias, effect sizes in single studies carried out with small samples might be substantially overestimated, meta- analyses—if also including studies reporting a lack of effects in very small samples—might underestimate efficacy. Therefore, it seems most appropriate to assume small to intermediate effect sizes (between *d* = 0.4 and *d* = 0.5 according to Cohen, 1988) when planning a tDCS study with healthy young participants and performing *a priori* power analysis. Moreover, we recommend open, accessible data so that small data sets can be potentially merged or analyzed using for example Bayes statistics.

## Author contributions

Each author contributed to designing the study, recording and analyzing the data, interpreting the results and writing the manuscript. BB and PS implemented the study.

## Funding

This research was funded by an LMUexcellent Investment Grant awarded to PS.

### Conflict of interest statement

The authors declare that the research was conducted in the absence of any commercial or financial relationships that could be construed as a potential conflict of interest.
